# Summary of Year-One Effort of the RCMI Consortium to Enhance Research Capacity and Diversity with Data Science

**DOI:** 10.3390/ijerph20010279

**Published:** 2022-12-24

**Authors:** Christopher S. Awad, Youping Deng, John Kwagyan, Abiel Roche-Lima, Paul B. Tchounwou, Qingguo Wang, Muhammed Y. Idris

**Affiliations:** 1 Department of Medicine, Emory University School of Medicine, 100 Woodruff Circle, Atlanta, GA 30322, USA; 2 Department of Medicine, Clinical Research Center, Morehouse School of Medicine, 720 Westview Dr SW, Atlanta, GA 30310, USA; 3 Department of Quantitative Health Sciences, John A. Burns School of Medicine, University of Hawaii at Manoa, Honolulu, HI 96813, USA; 4 Department of Community Health and Family Medicine, Howard University College of Medicine, 520 W St, Washington, DC 20059, USA; 5 Department of Bioinformatics, Medical Science Campus, University of Puerto Rico, CCHRD-RCMI, P.O. Box 365067, San Juan, PR 00936, USA; 6 Department of Biology, Jackson State University, 1400 J R Lynch Street, Jackson, MS 39217, USA; 7 Department of Computer Science & Data Science, School of Applied Computational Sciences, Meharry Medical College, 1005 Dr. D.B. Todd Jr. Blvd., Nashville, TN 37208, USA

**Keywords:** health disparities, training, data science, diversity, biomedical workforce

## Abstract

Despite being disproportionately impacted by health disparities, Black, Hispanic, Indigenous, and other underrepresented populations account for a significant minority of graduates in biomedical data science-related disciplines. Given their commitment to educating underrepresented students and trainees, minority serving institutions (MSIs) can play a significant role in enhancing diversity in the biomedical data science workforce. Little has been published about the reach, curricular breadth, and best practices for delivering these data science training programs. The purpose of this paper is to summarize six Research Centers in Minority Institutions (RCMIs) awarded funding from the National Institute of Minority Health Disparities (NIMHD) to develop new data science training programs. A cross-sectional survey was conducted to better understand the demographics of learners served, curricular topics covered, methods of instruction and assessment, challenges, and recommendations by program directors. Programs demonstrated overall success in reach and curricular diversity, serving a broad range of students and faculty, while also covering a broad range of topics. The main challenges highlighted were a lack of resources and infrastructure and teaching learners with varying levels of experience and knowledge. Further investments in MSIs are needed to sustain training efforts and develop pathways for diversifying the biomedical data science workforce.

## 1. Introduction

The National Institutes of Health (NIH) has had long-term strategic interests in bolstering the diversity of the biomedical and behavioral research communities [1]. While efforts have been marshaled by the NIH and other entities to invigorate the diversity amongst biomedical and behavioral research teams, diversity among funded investigators does not yet reflect the United States population [2,3]. Significant challenges also persist around the lack of representation from underserved communities in the biomedical data science workforce. Despite being disproportionately impacted by health disparities, Black, Hispanic, Indigenous, and other underrepresented populations account for 12% of graduates from doctoral programs in biomedical data science-related disciplines, a proportion that has not changed for the past 20 years [4]. Further down the pipeline, there is also a lack of diversity in faculty and mentors within and outside of biomedicine [5], both of which are essential for enhancing the participation of students and developing a talent pipeline of investigators from underrepresented groups in data science.

Data science has grown tremendously in the past several years due to its great potential to transform industries, economies, and scientific discoveries. Many data science initiatives from the NIH, e.g., All of Us Precision Medicine, Bridge to Artificial Intelligence (BRIDGE2AI), are largely focused on the generation of flagship data sets to accelerate health research and improve the health of racial and ethnic minority populations [6,7]. Although data science skills, ranging from best practices in data collection, labeling, and analysis to complex computational techniques like machine learning, are critical for advancing the science of minority health and health disparities [8], opportunities and the capacity for data science training have historically been limited at minority serving institutions (MSIs). To address this urgent issue, in May 2021 the National Institute for Minority Health Disparities (NIMHD) released a Notice of Special Interest (NOSI) entitled “Administrative Supplements to Enhance Data Science Capacity at NIMHD-Funded Research Centers in Minority Institutions (RCMI)”. This grant became the source of funding for the six awarded projects that we summarize here in this paper.

This paper aims to summarize all the NIMHD-funded RCMI institutions and their efforts with the development, deployment, and delivery of their respective data science training programs. We evaluate and highlight the reach, breadth, and feedback of these training programs with a particular emphasis on the targeted population and topics covered in the curriculum, as well as challenges & successes faced while designing and implementing these programs. Specifically, we utilized a cross-sectional survey to measure the demographics of learners served, curricular topics covered, methods of instruction and assessment, challenges, and recommendations by program directors. In what follows, we provide an overview of the RCMI program and the funding announcement from the NIMHD. We then provide an overview of survey questions and report on the results using descriptive methods. We conclude with a discussion on challenges and recommendations highlighted by project directors and link these results with the broader literature on developing and sustaining training programs with the stated goal of enhancing diversity.

## 2. Materials and Methods

The study design consisted of a cross-sectional survey administered at six minority-serving institutions awarded supplements from the NIMHD to develop new data science training programs. This included RCMI programs at the University of Puerto Rico—Medical Sciences Campus, Meharry Medical College, Jackson State University, the University of Hawaii at Manoa, Howard University, and the RCMI coordinating center at the Morehouse School of Medicine.

### 2.1. Research Centers at Minority Institutions (RCMI) Program

The Research Centers in Minority Institutions (RCMI) Program is a congressionally sponsored program initiated by the NIH in 1985 and administered by the National Center for Research Resources (NCRR) [9]. Imbedded within this legislation was the understanding that investments into building and maintaining research capacity at minority educational institutions was imperative to addressing health disparities at a national scale. The legislation used to establish the RCMI expected at least two-thirds of allocated funds to be used to build multi-user resources with the intention of building a network of resources amongst RCMI institutions.

Since its establishment, the RCMI program has proven itself as a research powerhouse, with extraordinary strength across the spectrum of translational medicine, health disparity research, and minority research participant enrollment. As noted by Ofili et al., between 2000 and 2015 RCMI investigators from 18 sponsored institutions leveraged $805 million in RCMI program funds into $3.7 billion in additional awards, including 1643 R01 (or equivalent) awards, with >14,000 publications and >500 patent disclosures [1].

### 2.2. Administrative Supplements to Enhance Data Science Capacity at NIMHD-Funded Research Centers in Minority Institutions (RCMI)

To address a lack of diversity in biomedical data science and to be better positioned for the challenges posed by the complex, highly dimensional, and inherently transdisciplinary nature of health disparities, the National Institute of Minority Health Disparities (NIMHD) issued a notice of special interest (NOSI) to invite applications for administrative supplements from eligible RCMIs. This funding opportunity is aimed at enhancing data science capacity at recipient institutions and fostering further collaborations between RCMI-funded researchers and data scientists [10].

The emphasis of the supplement was placed on the development of data science skill-building activities. In the notice, the activities of interest included the development of new modules focused on data analysis with health disparities datasets. Additionally, the NOSI cited interest in the integration of datasets representing “multiple levels and domains of influence in the NIMHD Research Framework” [11].

### 2.3. Survey Instrument

The survey consisted of 25 questions and focused on understanding the breadth of the curriculum marshaled at each program. They survey was modified to assess additional criterion, such as program-specific data, including demographics of trainees, knowledge areas covered in training programs, mechanisms for instruction, and assessed competencies. The questions in this survey were based on an ACM Data Science Task Force questionnaire administered to academic and industry participants to understand the landscape of curricular offerings in data science [12]. The survey also included open-ended response-type questions relating to challenges faced designing and implementing training programs and recommendations for enhancing diversity in biomedical data science were also collected in the survey from all participating institutions. Specific survey questions and types are demonstrated in Table A1. The survey was created and administered using Qualtrics software (Qualtrics, Provo, UT, USA).

### 2.4. Data Analysis

Descriptive analysis was performed on all quantitative survey data, including frequencies and percentages. Additionally, themes were identified in open ended questions around challenges and recommendations inductively.

## 3. Results

### 3.1. The RCMI NOSI Programs

Six NIMHD-funded RCMIs were awarded to increase data science capacity for resource-constrained, minority-serving institutions. These institutions included the Medical Sciences Campus at the University of Puerto Rico, Meharry Medical College, Jackson State University, the University of Hawaii at Manoa, Howard University, and the RCMI-Coordinating Center located at the Morehouse School of Medicine.

The specific aims of each of these training programs are detailed below:

University of Puerto Rico—Medical Sciences Campus: To enhance and build capacity for investigators and students from biomedical informatics and other disciplines in DS/AI/ML topics such as Jupiter Hub, coding with R, RStudio, and Python, using ML libraries and other cutting-edge techniques to mitigate Hispanics’ health disparities. To accomplish this aim, we developed of a new course (i.e., “Applying Artificial Intelligence and Machine Learning to Health Disparities Research (AIML+HDR)”), focused on data analysis using Hispanics datasets, that represents multiple levels and domains of influence in the NIMHD Research Framework.

Meharry Medical College: Develop authentic and sustainable collaborations between Meharry’s RCMI researchers and data scientists within Meharry’s new School of Applied Computational Sciences. Enhance Meharry’s RCMI capacity by providing data science training to the community. Assess the learning and data analytics skills of the RCMI investigators, post-docs, staff, and/or graduate students and research capacity enhancement of the RCMI program.

Jackson State University (JSU): With the overarching goal to train the next generation of scientists to address the monumental challenge associated with the curation, integration, analysis, and interpretation of biomedical big data, the specific aims of the Biomedical Data Science Training Program (BDS-TP) at JSU are to foster collaborations between data scientists and biomedical, socio-behavioral, and/or clinical researchers at the RCMI Center for Health Disparities Research at JSU; and build the capacity of JSU and other Historically Black Colleges and Universities (HBCUs) in Mississippi (Alcorn State University, Mississippi Valley State University, and Tugaloo College) in biomedical data science by developing the biomedical data science skills of science, technology, engineering, and mathematics (STEM) faculty, students, and post-doctoral research fellows.

University of Hawaii at Manoa: Provide education and training of data science-related skills to RCMI Researchers and their collaborators. Enhance collaborations between RCMI-funded researchers and data scientists. In addition to training RCMI investigators and their collaborators in a group in Aim 1, we will also offer individual data science training for investigators to foster collaborations between investigators and our data science experts.

Howard University: To develop a new and targeted intensive data science modular training workshop focused on data analysis with minority health and health disparities datasets. To develop hands-on experience in the utilization of data science in minority health and health disparity research, which includes: (a) develop mentored summer research projects for undergraduate, graduate, and post-doctoral trainees and, (b) host product driven Codeathon events to address an issue or issues related to minority health or health disparity research. To promote and support the institutionalization of data science efforts at Howard University by: (a) developing train-the-trainer programs that provide targeted training opportunities for HU educators and encourage them to incorporate data science principles and exposures in their class offerings; and (b) developing a train-the-researcher program that provides training opportunities for HU RCMI researchers with emphasis on junior faculty to effectively incorporate cutting-edge data science techniques and capabilities into their research efforts.

RCMI-CC at the Morehouse School of Medicine: To enhance the data science capacity of RCMI investigators across the RCMI consortium by collaboratively developing a project-based curriculum and organizing workshops introducing data science fundamentals and tools, as well as NIH-funded resources and datasets relevant to minority health disparities. To stimulate data science collaborations across U54 centers, as well as between U54 and non-RCMI institutions by organizing Data Science and Health Disparities Demo Days and launching enrichment awards to support the development of collaborative research projects.

### 3.2. Data Science Trainees

Each program recruited a diverse cohort of trainees at differing stages in their careers, ranging from undergraduate students to postdoctoral fellows and early-stage investigators and senior faculty (see Table 1). The participants’ research backgrounds were almost evenly split amongst basic science, clinical/translational, and social/behavioral research (see Table 2). Across all the programs, there was also diverse demographic participation and almost equal male and female participation (see Table 3). 

### 3.3. Data Science Curriculum

**Figure 1:**
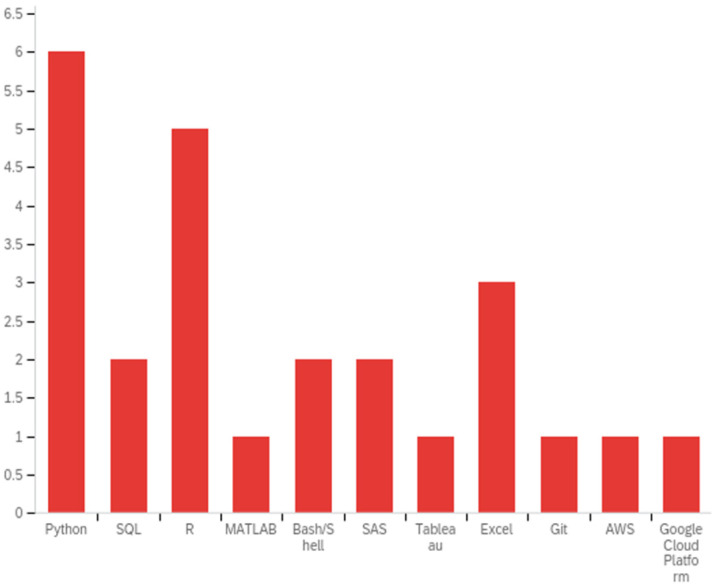
Tools covered by training program curriculum (count of programs; q13).

Table 4 offers a look at the breadth of curricular offerings by program. Almost all institutions offered more than one computer programming course as a part of their curriculum. When surveyed about concepts and tools offered to students, the programs demonstrated an impressive breadth of topics. Of the twenty concepts in computing knowledge surveyed across program curriculums, only two were not offered (i.e., web development and data privacy and security) at any program. Figure 1 presents counts of the data science tools that were covered in curriculum as a count.

### 3.4. Data Science Instruction

Institutions utilized existing resources, created new resources, and invited instruction from outside faculty (see Table 5). Funded programs were accessed by learners with increasing options for asynchronous learning (as shown in Table 6), with most programs opting to offer synchronous program times. Similarly, students were assessed by a variety of means, with only one program giving their students no homework or assessments (see Table 7).

### 3.5. Programmatic Challenges

Survey respondents were asked to enumerate the top three challenges faced when designing and implementing their training programs (Appendix A; Question #16). Those findings are summarized in Table 8, which aims to succinctly share overlapping themes in respondents’ feedback. Broadly, respondents shared that creating programming with representative datasets was made difficult because of a lack of resources and infrastructure. This is in addition to identifying and recruiting internal and external faculty or staff to lead instruction. Similarly, there seemed to be a shared challenge amongst respondents who were attempting to create programming that could be used by a broad audience with varying levels of technical experience and diverse lived experiences.

### 3.6. Recommendations from RCMI Program Coordinators

For the last question of the survey, the respondents were asked to share recommendations for enhancing diversity in biomedical data science (BDS), generally. The responses were varied but focused on similar themes, summarized in Table 9. Reflecting the challenges (summarized in Section 3.5), the respondents broadly pointed to issues regarding resource and (infra)structure development. Notably, the respondents took a long-term approach to their recommendations, citing not only the need to build long-lasting support and training for future RCMI programs, but also long-lasting systems for participation and models of engagement.

Examples of these recommendations were ‘training-the-trainer’ models of didactics, embedded multi-level coursework inside training programs (to allow beginners and experts to benefit from the same resources), and investments in diverse faculty and mentorship to broaden the interdisciplinary reach at partnering institutions.

## 4. Discussion

The purpose of this study was to shed light on the reach, curricular breadth, and best practices in data science training at resource-constrained, minority-serving institutions by summarizing the efforts of six NIMHD-funded RCMI programs that received funding to develop data science training programs. The programs surveyed shared curricular goals around the application of cutting-edge data science techniques to different data types and training development around data science resources and tools. The average program used four tools and discussed over ten (>10.6) computing knowledge areas over the course of their curriculum. Together, the programs targeted building capacity at every point in the higher-education process (from undergraduates to continuing education for professors). The shared goals of the RCMI programs were to increase training capacity, professional development, and institutional collaboration. These goals have been highlighted across several RCMI initiatives and in the evaluation of RCMI programming [13,14,15].

The survey results also emphasize several pertinent themes around challenges in, and recommendations for, designing, implementing, and sustaining training programs to enhance diversity in data science training. Notably, the challenges and recommendations were reflective of issues of long-term planning and investment. For instance, the need for broad infrastructure was a recurring theme in survey responses; the possibilities for infrastructure development to bridge the chasm in data generation, analysis, access, and distribution has been echoed for several years [8,16,17]. Additional challenges included the inability to create curricula that reflects the needs of their audiences due to the lack of minority-relevant data sets and the challenges recruiting a diversity of instructors. Others have written extensively about the lack of diversity in STEM, which remains a challenge for the US biomedical workforce [5], but the lack of minority-relevant data sets is a novel insight and likely speaks to a growing call for representation in training data as a basis for fair/unbiased data science models being applied in health disparities research [18,19].

While the recommendations were diverse, the most agreed upon recommendations can be summarized by the prioritization and development of long-lasting resources. Program directors independently recognized the ways that diverse data set development, training for learners at various levels of erudition, and a multiplicity of training options (e.g., domains, tools) would provide a better outlook for future generations of diverse investigators with robust data science skills. A plethora of educational modalities for healthcare researchers at various stages of education have demonstrated comparable performance and increased the reach of educational objectives [20,21,22] and diversity of participants when programs are available and best practices are adhered to [23,24,25].

### Limitations

There are three limitations of our study that are worth considering: first, the six RCMI programs considered are not a random sample of RCMI institutions. The programs covered were awarded grants through a competitive process, which limits the generalizability of our findings. Second, the funding opportunity from the National Institute for Minority Health disparities did not require a formal evaluation plan. As such, the programs evaluated did not utilize the same approaches to evaluating their respective programming. While our analysis attempted to develop a better understanding of who took part and what would be required to sustain the program over time, future studies should incorporate a formal evaluation of the data science programs using an existing framework like RE-AIM [26]. Finally, it is worth noting that our study focused on participants who, while minorities, are already living in a high-income country, and may not be generalizable to resource-constrained environments that exist in lower-income settings.

## 5. Conclusions

This article adds to the literature on investments in minority-serving institutions with the twin aims of enhancing capacity in these resource-constrained environments and contributing to the diversification of the scientific workforce. Our descriptive study provides insight on federally funded data science training programs leveraging the breadth and depth of the NIMHD-funded RCMI network. Our results support the need for increased diversity in data sets, increased curricular materials and work sets reflective of the diverse audiences served, and further diversity of personal and professional exposure for underrepresented professionals in biomedical data science. Investment and funding efforts to build and sustain increased capacity at RCMI and minority-serving institutions are necessary and may prove increasingly so as the diversity of the nation continues to outpace the diversity reflected in NIH-funded datasets and research. Taken together, there is strong evidence for building capacity in biomedical data science at RCMI institutions and the downstream impact for enhancing diversity in the biomedical research and data science workforce. 

## Figures and Tables

**Table 1:** Demographic Targets of all participating programs.

Program Name	UPR	Meharry Medical College	Jackson State University	Hawaii	Howard	RCMI-CC
Undergraduate Students				✓		
Graduate Students	✓	✓	✓	✓	✓	✓
Instructors	✓	✓		✓	✓	✓
Assistant Professors	✓	✓	✓	✓	✓	✓
Associate Professors	✓		✓	✓	✓	✓
Full Professors	✓	✓		✓	✓	✓
Post-Docs/Fellows		✓	✓	✓	✓	✓
Others		✓				

✓ indicate that the institution participated in the programmatic or curricular header in each table.

**Table 2:** Type of Research conducted by participants.

	%	Count
Basic	36.84%	7
Clinical and/or Translational	31.58%	6
Social/Behavioral Science	31.58%	6
Other [Specify]	0.00%	0

**Table 3:** Demographic Summary of participants served through programmatic efforts.

Participating Institutions	UPR	Meharry	Jackson State	Hawaii	Howard	RCMI-CC
Male/Female	62/38	40/60	40/60	55.5/44.5	n/a	42.96/57.04
Black	0%	>50%	48%	22.6%	n/a	32.59%
Asian	0%	<30%	40%	30.2%	n/a	21.48%
Hispanic/Latina/o	100%	n/a	0%	10.5%	n/a	28.89%
Hawaiian/Pacific Islander	0%	n/a	0%	17.2%	n/a	2.96%
Native American	0%	n/a	0%	2%	n/a	0%
White	0%	n/a	8%	20.4%	n/a	10.37%
Other	0%	n/a	0%	3.8%	n/a	3.7%

**Table 4:** Summary of curricular topics covered.

	UPR	Meharry	Jackson State	Hawaii	Howard	RCMI-CC
Python	✓	✓	✓	✓	✓	
R	✓	✓	✓	✓		✓
AWS	✓					
SQL		✓	✓			
BASH/SHELL		✓		✓		
Tableau			✓			
Excel		✓	✓	✓		
Google Cloud				✓	✓	
Git		✓				
SAS		✓	✓			
MATLAB				✓		

✓ indicate that the institution participated in the programmatic or curricular header in each table.

**Table 5:** Summary of program instruction.

Program Name	New Courses	Workshops/Seminars by Existing Faculty	Workshops/Seminars by Outside Faculty	Existing Courses	Asynchronous Options
UPR	✓	✓	✓		✓
Meharry		✓	✓	✓	
Jackson State		✓	✓		
Hawaii		✓	✓	✓	
Howard		✓	✓		✓
RCMI-CC		✓	✓		

✓ indicate that the institution participated in the programmatic or curricular header in each table.

**Table 6:** Count of programs offering asynchronous options (q14).

Answer	%	Count
Yes	12.50%	1
No	50.00%	4
We recorded our lectures and will be releasing them async	37.50%	3
Total	100%	8

**Table 7:** Summary of program assessments.

Program Name	Use of Assessments	Use of Projects	Presentations	Assignments (Homework)	No Assessments
UPR	✓	✓			
Meharry	✓	✓	✓	✓	
Jackson State	✓				
Hawaii	✓	✓	✓		
Howard		✓		✓	
RCMI-CC					✓

✓ indicate that the institution participated in the programmatic or curricular header in each table.

**Table 8:** Summary of challenges faced designing and implementing programming.

	Program Name					
	UPR	Meharry	Jackson State	Hawaii	Howard	RCMI-CC
Topic/Scope	✓					
Finding appropriate data sets	✓	✓				✓
Recruiting Internal Faculty/Staff			✓			✓
Recruiting External Faculty/Staff	✓				✓	✓
Infrastructure		✓	✓			
Funding				✓		
Scheduling		✓	✓		✓	✓

✓ indicate that the institution participated in the programmatic or curricular header in each table.

**Table 9:** Summary of recommendations by program directors.

	UPR	Meharry	Jackson State	Hawaii	Howard	RCMI-CC
Training for under-represented populations	✓				✓	
Diversity data sets	✓	✓				✓
Infrastructure development			✓			✓
Training for learners of various levels	✓				✓	✓
Diverse Faculty Recruitment		✓	✓			
Sustainable Funding				✓	✓	
Synchronous & Asynchronous options		✓	✓			✓

✓ indicate that the institution participated in the programmatic or curricular header in each table.

## Data Availability

The publicly archived datasets analyzed in the study can be found here: https://doi.org/10.7910/DVN/UG4OM7 (accessed on 1 December 2022).

## Appendix A

**Table A1:** Survey Questions Administered to Program Coordinators at Participating RCMI Programs.

Question #	Survey Question	Type of Question
1	Please select your institution/program	[Multiple select]—Selected Choice
2	What is the stage of your target participants?	[Multiple select]—Selected Choice
3	What is the stage of your target participants?	[Multiple select]—Other: Please Enter—Text
4	What type of research do your participants conduct?	[Multiple select]—Selected Choice
5	What type of research do you conduct?	[Multiple select]—Other [Specify]—Text
6	What percentage of your participants identified as...Male	[Numeric]
7	What percentage of your participants identified as...Female	[Numeric]
8	What percentage of your participants identified with the following Ethnicity/Race categories...African American (Black)	[Numeric]
9	What percentage of your participants identified with the following Ethnicity/Race categories...Asian	[Numeric]
10	What percentage of your participants identified with the following Ethnicity/Race categories...Hispanic or Latina/o	[Numeric]
11	What percentage of your participants identified with the following Ethnicity/Race categories...Hawaiian Pacific Islander	[Numeric]
12	What percentage of your participants identified with the following Ethnicity/Race categories...Native American	[Numeric]
13	What percentage of your participants identified with the following Ethnicity/Race categories...White	[Numeric]
14	What percentage of your participants identified with the following Ethnicity/Race categories...Other	[Numeric]
15	Which of the following (computing) knowledge areas did your training program cover	[Multiple select]—Selected Choice
16	Which of the following (computing) knowledge areas did your training program cover	[Multiple select]—Other—Text
17	Which of the following tools did your training program cover	[Multiple select]—Selected Choice
18	Which of the following tools did your training program cover	[Multiple select]—Other—Text
19	How were participants being taught?	Selected Choice
20	How were participants being taught?	Other—Text
21	Did you offer any asynchronous options?	[Open Ended]
22	How were competencies assessed?	Selected Choice
23	How were competencies assessed?	Other—Text
24	What are the top 3 challenges you faced designing and implementing your training program	[Open Ended]
25	What are that 3 recommendations do you have for enhancing diversity in biomedical data science	[Open ended]
